# Improving hospital-based end of life care processes and outcomes: a systematic review of research output, quality and effectiveness

**DOI:** 10.1186/s12904-017-0204-1

**Published:** 2017-05-19

**Authors:** Amy Waller, Natalie Dodd, Martin H. N. Tattersall, Balakrishnan Nair, Rob Sanson-Fisher

**Affiliations:** 1 Priority Research Centre in Health Behaviour, University of Newcastle, Callaghan, NSW 2308 Australia; 2 Hunter Medical Research Institute, Newcastle, NSW 2305 Australia; 3 University of Sydney, Chris O’Brien Lifehouse, Level 6 North, Missenden Road, Camperdown, 2050 Australia; 4 Faculty of Health and Medicine, University of Newcastle, Callaghan, NSW 2308 Australia; 5 Hunter New England Local Health District, Newcastle, 2305 Australia

**Keywords:** End-of-life, Hospital, Advance care planning, Palliative care, Acute care

## Abstract

**Background:**

As in other areas of health delivery, there is a need to ensure that end-of-life care is guided by patient centred research. A systematic review was undertaken to examine the quantity and quality of data-based research aimed at improving the (a) processes and (b) outcomes associated with delivering end-of-life care in hospital settings.

**Methods:**

Medline, EMBASE and Cochrane databases were searched between 1995 and 2015 for data-based papers. Eligible papers were classified as descriptive, measurement or intervention studies. Intervention studies were categorised according to whether the primary aim was to improve: (a) end of life processes (i.e. end-of-life documentation and discussions, referrals); or (b) end-of-life outcomes (i.e. perceived quality of life, health status, health care use, costs). Intervention studies were assessed against the Effective Practice and Organisation of Care methodological criteria for research design, and their effectiveness examined.

**Results:**

A total of 416 papers met eligibility criteria. The number increased by 13% each year (*p* < 0.001). Most studies were descriptive (*n* = 351, 85%), with fewer measurement (*n* = 17) and intervention studies (*n* = 48; 10%). Only 18 intervention studies (4%) met EPOC design criteria. Most reported benefits for end-of-life processes including end-of-life discussions and documentation (9/11). Impact on end-of-life outcomes was mixed, with some benefit for psychosocial distress, satisfaction and concordance in care (3/7).

**Conclusion:**

More methodologically robust studies are needed to evaluate the impact of interventions on end-of-life processes, including whether changes in processes translate to improved end-of-life outcomes. Interventions which target both the patient and substitute decision maker in an effort to achieve these changes would be beneficial.

**Electronic supplementary material:**

The online version of this article (doi:10.1186/s12904-017-0204-1) contains supplementary material, which is available to authorized users.

## Background

Between one-third and two thirds of people in developed world countries will die in hospital, and approximately 20% of people will die in an intensive care unit [1, 2]. People living longer with chronic diseases and limited availability and access to well-resourced community services have contributed to the increasing trend for institution-based deaths [3, 4]. However, many people either do not understand or are unaware of end-of-life care options [5]. Health care providers involved in the care of dying patients report difficulties in knowing when and how to withdraw or withhold life-sustaining treatments [5, 6]. Dying in hospital has been associated with high rates of unwanted aggressive treatment, underuse or late use of palliative care and poorer symptom management [7–10].

Process and consequences of health delivery are important aspects of care to measure. Processes include those things that are in immediate control of healthcare providers and are intended to improve the outcomes associated with end-of-life care, such as goals of care discussions; end-of-life documentation (e.g. advance care directives (ACDs), do-not-resuscitate (DNR) orders); involvement of support persons in decision-making; and referrals to hospice. End-of-life outcomes are seen as functions of the processes of care patients undergo and the structures in which these processes occur (e.g. hospital, skill mix). End-of-life outcomes may include perceived health status, quality of life; concordance between preferred and actual care; survival; and costs or utilization. It is expected that successful implementation of end-of-life processes will be associated with improved end-of-life outcomes.

A number of approaches are hypothesised as a means of improving end-of-life processes and outcomes, including advance care planning, family meetings and palliative care consultations. Previous systematic reviews have synthesised the literature across a range of care settings for certain interventions, such as ACP [11, 12]. Others have focused on the impact of different interventions in care settings, such as the intensive care unit (ICU) [13, 14]. There has been limited synthesis of the evidence pertaining to the impact of these interventions on end-of-life processes and outcomes in general hospital settings. This is an important gap for a number of reasons. First, the rise in the number of deaths occurring in this setting in many countries is likely to place increasing pressure on already finite resources, which may result in suboptimal care [3, 15]. Second, there are high personal and societal costs associated with suboptimal end-of-life care, highlighting the need for improvements. Third, the success of interventions may be dependent on the environmental context in which they are applied [16]. Interventions successfully applied to stable outpatients or in the general community may not achieve similar improvements in hospital.

Given the limited health service resources available, it is important that end of life care is evidence-based, rather than based solely on the intuition of service providers. Research must meet minimum standards of scientific quality to ensure adequate internal and external validity. [17]. Measurement studies involve the development of psychometric tools that can reliably and accurately assess end-of-life processes or outcomes. Such tools are used to provide empirical data describing the prevalence and correlates of the outcomes and inform how we might intervene to address important gaps in care. Intervention studies can provide evidence of effective strategies that can be implemented to reduce gaps. The quality of the studies must also be established; as high volume doesn’t necessarily equate with quality. Despite potentially adverse consequences of suboptimal hospital-based end-of-life care, the quality, relevance and impact of research associated with end-of-life processes and outcomes in hospitals has not been examined.

## Methods

**Aims**: This systematic review aimed to examine the:

1. volume and type of data-based publications examining end-of-life care among people dying in hospital and their families;
2. methodological quality of intervention studies aimed at improving end-of-life processes and outcomes according to EPOC methodological criteria; and
3. the effectiveness of interventions in studies that met this criteria.

### Search strategy

**Fig. 1:**
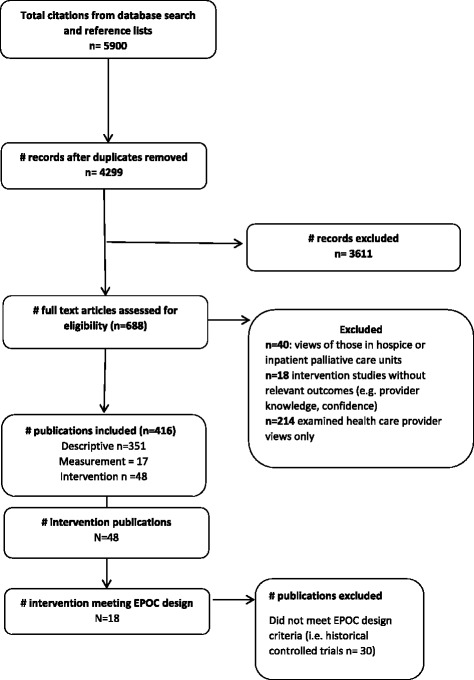
Search strategy

A search of MEDLINE, EMBASE, and CINAHL databases was conducted by one author (AW) and a medical librarian independently (see acknowledgements) based on the search strategy in Fig. 1, limited to articles published between 1995 and December 2015. The search strategy for each of the databases is outlined in Additional file 1. Searches were restricted to human studies published in English.

### Inclusion and exclusion criteria

Studies were included if they focused primarily on:

- end-of-life processes: end-of-life/goals of care discussions; end-of-life documentation (e.g. ACDs, DNR orders); appointment of substitute decision makers; medication orders; or referrals to hospice/palliative care; and/or
- end-of-life care outcomes: health status, satisfaction and quality of life; perceived quality of care; concordance of preferred and actual care; survival; or health care costs or utilization;
- Studies examined these outcomes among adults (18 years or over) admitted to hospitals (excluding intensive care units) or their families.

Studies were excluded if they were book chapters, review articles, case studies, commentaries, conference abstracts, editorials or protocol papers.

### Data coding

Paper titles were initially assessed against the eligibility criteria by AW and excluded if the study did not meet inclusion criteria. A random sub-sample (20%) of included and excluded studies were categorised by another author (ND), with any discrepancies resolved via discussion. Papers were then categorized as either:

***Measurement studies*** included those describing the development or testing of the psychometric quality of tools to assess either end-of-life care processes or outcomes.

***Descriptive studies*** documented frequency, patterns, correlates and/or preferences in relation to either end-of-life care processes or outcomes using quantitative or qualitative methods.

***Intervention studies*** were categorised into two group: (1) those where the primary aim was to examine the impact of interventions on end-of-life processes; or (2) those where the primary aim was to examine the impact of the intervention on end-of-life outcomes.

### Assessment of methodological quality

Intervention studies were assessed as to determine whether the experimental design was one of the four types allowed by the EPOC design criteria - randomized controlled trials, controlled trials, controlled before and after studies, or interrupted time series studies [18] Stepped wedge designs were also included as they are a viable alternative to a parallel cluster randomised trial and accepted by EPOC as a robust design. For those studies meeting minimum design criteria, methodological quality was then assessed using EPOC risk of bias criteria independently by two reviewers (AW and ND).

### Assessment of effectiveness

Additional study data was extracted from each intervention study that met the minimum criteria for quality, including: aim; study setting; sample characteristics; inclusion and exclusion criteria; intervention design; outcome measures; follow-up periods and study findings.

### Analysis

Poisson regression was used to model trends over time in the numbers of publications. Percent change by year with Wald 95% confidence are presented. *P*-values were calculated from the Wald Chi-square.

## Results

### Search results

A total of 4611 were identified for potential inclusion, after removal of duplicates. After assessment against eligibility criteria, 416 publications met criteria for inclusion in the review. A flow chart of the literature search and paper identification is provided in Fig. 1.

### Number and type of published studies of end-of-life care in hospitals (1995–2015)

**Fig. 2:**
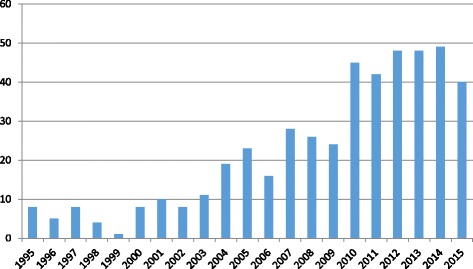
Number of publications by year

Poisson regression shows the number of publications increasing by 13% each year (95%CI = 11.1–11.5%; *P* < 0.0001) (see Fig. 2). The majority of eligible studies were descriptive studies (*n* = 351, 85%). Of these, 145 were descriptive studies describing the views of patients or carers (*n* = 145); and 206 were medical record audits. There were 17 measurement studies; with the remaining 48 studies reporting on interventions. Only 18 studies met EPOC design criteria (Table 1). Of these, 11 focused on end-of-life processes as their primary outcome [19–29]; and seven focused on end-of-life outcomes as their primary outcome [30–36] (Additional file 2).

**Table 1:** Quality of intervention studies meeting EPOC design criteria (Low, High, Unclear)

Author, Date Design	Type	Allocation sequence adequately generated?	Concealment of allocation	Baseline outcome measurement similar	Baseline characteristics similar	Incomplete outcome data adequately addressed	Knowledge of allocated interventions prevented	Protections against contamination	Selective outcome reporting	Free other risk of bias
End of life outcomes										
Costantini 2014 [31]	CRCT	L	L	L	L	L	H	L	L	H
Detering 2010 [36]	RCT	L	L	L	L	L	L	H	L	L
Gade 2008 [32]	RCT	L	L	L	H	L	U	U	L	L
Hanks 2002 [33]	RCT	L	L	L	H	L	U	U	L	L
Sidebottom 2015 [34]	RCT	U	U	L	H	L	U	H	L	L
Song 2005 [35]	RCT	U	U	L	L	L	U	U	L	L
The SUPPORT Principal Investigators 1995 [30]	RCT	L	L	L	L	L	L	U	L	L
End of life processes										
Ahronheim 2000 [29]	RCT	U	U	L	L	L	L	H	L	H
Bailey 2014 [19]	SW	H	L	L	L	L	L	L	L	L
Cugliari 1995 [20]	CCT	H	H	U	H	U	U	H	L	L
El-Jawahri 2015 [21]	RCT	L	L	L	L	L	H	U	L	L
Grimaldo 2001 [22]	RCT	L	L	L	L	L	U	U	L	L
Jacobsen 2011 [23]	CCT	H	H	U	L	L	H	L	L	L
Meier 1996 [24]	RCT	U	U	U	L	L	U	U	L	L
Nicolasora 2006 [25]	RCT	L	U	U	L	L	H	U	L	L
Sampson 2011 [27]	RCT	U	U	L	L	L	U	L	L	L
Study (ITS)	Independent of other changes?		Shape of effect pre-specified?	Intervention affected data collection?	Knowledge interventions adequately prevented	Incomplete outcome data adequately addressed?		Free from selective outcome reporting?	Other risk of bias?	
Reilly 1995 [26]	L		L	L	L	L		L	U	

### Methodological quality of studies

Studies included a cluster randomised controlled trial [31]; a stepped wedge trial [19]; randomised control trials [17, 20–22, 24, 25, 27, 29, 30, 32–36]; controlled clinical trials [20, 23] and an interrupted time series trials [26] (Table 1). Five studies were rated as low risk on at least seven of the nine criteria. The most poorly met criteria included: not specifying whether outcomes were assessed blindly or protected against contamination. Studies did not report on either method of generating allocation sequence or concealing allocation.

### Effectiveness of intervention studies meeting EPOC design criteria

Table 2 presents the study characteristics of 18 intervention studies which were rated as high quality when compared to the EPOC criteria. Almost all of these studies were conducted in the USA [19–26, 28–30, 32, 34, 35], with one in the UK [27], one in Italy [31] and one in Australia [36]. Half of the studies targeted mixed seriously ill populations [19, 28, 29, 32, 33]. The remainder targeted the elderly [24, 36], surgical [22, 35], dementia [27, 29], heart failure [34]. Others included any admitted patient [20, 23, 26]. Three studies tested patient-directed interventions involving the provision of written information or audio-visual information [20, 21, 25]. Seven involved facilitated ACP interventions [22, 24, 27, 28, 30, 35, 36] and four were palliative care consultations [32–34, 29]. Four studies tested multi-faceted, system-based interventions [19, 23, 26, 31].

**Table 2:** Summary of findings of intervention studies (*n*=18)

Reference, Country Design	Sample & Setting	Inclusion & Exclusion	Intervention	Outcome measures and time points	Findings
End of life processes					
Ahronheim et al. 2000 [36] USA RCT	Sample: 99 Response rate: Setting: Hospital Diagnostic group: Advanced dementia Mean age: 84.8 (63–100)	Inclusion: FAST ≥6d, stable at least one month.	Type: Palliative care consultation, discussion with primary care team, family meeting and care recommendations Control: primary care team only	Primary: Mortality, site discharge, length of stay, readmissions DNR orders, systemic antibiotics Secondary: Decision to forgo treatments, decision to adopt palliative care plan. Follow-up: Discharge or in-hospital death	No differences in mortality, readmissions, length of stay. Intervention patients discharged more often with palliative care plan; and more decisions to forgo treatments
Bailey et al. 2014 [19] USA Stepped wedge	Sample: 6066 Response Rate: Setting: 6 VAMSs Diagnostic group: Veterans who died in centres – any illness Mean age: 71.2 (SD=12)	Staff: 1621 physicians, nurses, residents, allied health, pharmacy, mental health, admin and other Patients: all who died in 6 year period; and subset all who died in 12 month pre and 12 month post-intervention.	Type: Site visits, staff education, decision support tool (CCOS), follow-up consultations during 4 month training period Control: Retrospective chart audit	Primary: % with: Opioid order, DNR order, Location death; Nasogentric tube, IV line infusing, Restraints Secondary: Antipsychotic (order, given) Opioid given, Death rattle medication Benzodiazepine (order, given), PC consult, Pastoral visit, Advance directive, Sublingual admin Follow-up: 12 month-post intervention	Improved orders for opioid, antipsychotic medication, benzodiazepines, death rattle medication, and advance directives. Intervention effects were modest but statistically significant.
Cugliari et al. 1995 [20] USA CCT	Sample: 419 Setting: 2 hospitals Diagnostic group: mixed Mean age: 53 (SD=16.6)	Inclusion criteria: >18 years, planned admission Exclusion criteria: emergency, obstetrics, psychiatric admission, insufficient English	Type: Written information + 18 min videotape of interviews with adults about experience with advance care planning; and instructions on completing ADs. Control: Written information (usual care) on law on advance directives and health care proxy.	Recall of information Attitudes about ADs and decision to complete proxy	No difference between the groups in recall, understanding of proxy form, completion of form, or perceived importance of ADs. No difference in intention to complete form later.
El-Jawhari et al. 2010 [21] USA RCT	Sample: 150 Setting: Inpatient internal medicine in two hospitals Diagnostic group: Mixed Mean age: 76 (SD=13)	Inclusion criteria: >60 years, ability to provide consent; communicate in English; advanced cancer, heart failure, COPD, other advanced illness or prognosis <12 months.	Type: 3 minute video on CPR and intubation Preferences for CPR and intubation communicated to 1+ physician Control: Asked for CPR and intubation preferences only	Primary: CPR and intubation preferences Secondary: CPR/intubation orders, documented discussions with providers, patient knowledge CPR/intubation Follow-up: Post-video	Intervention patients more likely not to want CPR and intubation; have documented orders for CPR and intubation; documented discussions of preferences; and higher mean knowledge scores
Grimaldo et al. 2001 [22] USA RCT	Sample: 185 Setting: Pre-operative evaluation clinic Diagnostic group: scheduled for elective surgery Median age: 73.3	Inclusion criteria: English speaking; ≥65 years old; scheduled for elective surgery or overnight stay	Type: Usual care + 5–10 minute anaesthetist led information session focusing on the importance of patient-proxy communication about EoL care. Asked if had an AD and offered DPOA paperwork. Control: Standard pre-operative screening and counselling.	Primary: Increased dialogue between pt and proxies in clinic Follow-up: Pre- and post-operatively	Intervention group: Greater proportion who had DPOA at post-operative. More changed response no to yes about DPOA discussions. More likely to have discussions about medical care with proxy
Jacobsen et al. 2011 [23] USA CCT	Sample: 899 Setting: Two general medical wards Diagnostic group: general medicine, seriously ill Mean age: 63.5	Inclusion criteria: stable and unstable seriously ill patients admitted to general medicine ward.	Type: Stable pts: patient, family and provider meet to assess knowledge, preferences and experiences. Unstable pts: provider an family meet to recommend about treatment and prognostication. Control: Usual care not specified	Primary: % pts admitted full code without discussion documented % pts admitted full code with discussion documented % pts ACP discussion and order of WLST documented Follow-up: At discharge (record review)	Intervention ward significant better across all outcomes.
Meier et al. 1996 [24] USA RCT	Sample: 190 Setting: Geriatric inpatient unit Diagnostic group: Elderly Mean age:	Inclusion criteria: >65 years, met Medicare Prospective Payment guidelines, complex care problems	Type: Counselling about advance directives and provided opportunity to complete health care proxy, charting of advance directives and proxy forms. Control: Usual care	Primary: Documentation: (a) copy of proxy form; (b) patient proxy recorded; (c) advance directive notation Secondary: Self-reported quality of care Follow-up: One month	Intervention more likely to complete new proxy or have previously completed proxy identified.
Nicolasora 2006 [25] RCT	Sample: 297 Setting: Medical ward of teaching hospital Diagnostic group: Mixed Median age: 65	Exclusion criteria: cardiac catheterization or admission to ICU; documented dementia or delirium (control); of judged by physician to have impaired cognitive function (intervention)	Type: Script about CPR, mechanical ventilation delivered by physician; asked about CPR status; changes communicated to physician; wishes to prepare ADs and assisted with completing ADs Control: Medical records surveyed for in-hospital outcomes and AD status	Primary: Willingness to listen to script; acceptability of information; frequency of changing or choosing CPR status; rate of completion of ADs Secondary: Follow-up: Post-script	98% willing to discuss CPR and 82% useful 36% intervention had documented code status at admission (vs 34% control) 92% intervention clarified preferences 13/102 without ADs created one after intervention (vs 1/128 control)
Reilly et al. 1995 [26] USA ITS	Sample: 1780 Setting: Hospital Diagnostic group: mixed	Inclusion criteria: Not reported Patients and staff	Type: Education phase (Reminders, education and feedback to providers); Intervention phase (Standardised AD documentation form placed in medical charts) Control: Not specified	Primary: Frequency and content of ADs documented in charts; Secondary: Pt attitudes about ADs; Provider attitudes about ADs Follow-up: 10 time points (4 control, 3 education and 3 intervention)	Proportion ADs highest during intervention phase (63% vs 23%E vs 25% C) Frequency of ICU ADs and CPRs greater during intervention phase 87% ADs concordant with pt preferences
Sampson et al. 2011 [27] UK RCT (pilot)	Sample: 33 Response Rate: Setting: Two acute medical wards Diagnostic group: Advanced primary dementia Mean age: 59 (SD=13)	Inclusion criteria: Unplanned admission for treatable acute illness; presence of surrogate that was able to provide informed consent. (FAST stage 6d or worse)	Type: Component 1: 30 minute pt assessment and formulation of management plan. Component 2: Consultations with carers to discuss pts current situation and to provide education about dementia, ACP and PC (these occurred in hospital and in the community post d/c). Control:	Primary: No. carers with ACP Secondary: Carer measures: Distress (KD-10); health status QoL (EQ-5D); Decision making (DCS); decisional satisfaction (DSI); Anger (SAS); Life satisfaction (LSQ); EoL satisfaction (SWC-EOLCD). Patient measures: Pain, distress Follow-up: Patient- 6 weeks; 6 months. Carer-3 months after death.	Seven ACP’s were made in the I group Attrition precluded statistical comparison of groups
Teno et al. 1997 [25] USA	Sample: 4804 Response Rate: Setting: Five teaching hospitals. Diagnostic group: acute respiratory failure; multiple organ failure with sepsis or malignancy; coma; COPD; CHF; cirrhosis; metastatic colon cancer; NSCLC Mean age: 62 (SD=16)	Inclusion criteria: Presence of diagnosis and ≥18 years of age. Exclusion criteria: d/c or died <48 hours; admitted scheduled d/c <72 hours; non-English speaking; psychiatric ward; AIDS diagnosis; pregnancy; acute burns, trauma.:	Type: Connors et al. + PSDA mandated pt education and documentation of AD’s Control: Usual care not specified	Primary: Awareness, completion and documentation of AD’s; effectiveness of AD’s on decision-making about resuscitation; Physicians role in using AD’s; Surrogates perspectives of AD’s Secondary: Follow-up: 6 months.	Increase in AD documentation in the Post/I group, otherwise the intervention did not affect the pt familiarity with or the use of AD’s.
End of life outcomes					
The SUPPORT Principal Investigators 1995 [30] (SUPPORT) USA	Sample: 4804 Setting: Five teaching hospitals. Diagnostic group: acute respiratory failure; multiple organ failure with sepsis or malignancy; coma; COPD; CHF; cirrhosis; metastatic colon cancer; NSCLC	Inclusion criteria: Presence of diagnosis and ≥18 years of age. Exclusion criteria: d/c or died <48 hours; admitted scheduled d/c <72 hours; non-English speaking; psychiatric ward; AIDS diagnosis; pregnancy; acute burns, trauma.:	Type: Nurse led intervention: provision of prognostic information to improve communication and decision making. Pt and family EoL preferences elicited and documented. Control: Usual care not specified	Primary: Timing of DNR orders; Pt/physician concordance CPR preferences; Days in ICU before death; Pain; Hospital resource use Secondary: Follow-up: Audit on Days 1, 3, 7, 14 and 35.	Small improvement in Pt/physician concordance in the group. Small increase in reported pain.
Costantini et al. 2014 [31] Italy CRCT	Sample: 308 Setting: 16 general medicine wards Diagnostic group: Oncology patients and families Mean age:75.6 (SD=10.8)	Inclusion criteria (wards): 25+ cancer deaths per year, hospital consent, specialist palliative care team. Patients who died from cancer and their family member	Type: Liverpool care pathway; training of ward staff and palliative care unit staff; leaflets for family members on emotional and practical issues; audits and feedback; documentation. Control: Usual care	Primary: Overall mean score on toolkit after bereavement interview Secondary: Decision making, ACP, respect, emotional support, coordination care, self-efficacy, quality of care, control of symptoms, processes of care Follow-up: 2–4 months post-bereavement (family); processes 6 months post-implementation.	No difference in overall rating of care. Improvements in 2/9 secondary outcomes only (respect, and control of breathlessness) No difference in survival or medication prescribed
Detering et al. 2010 [36] AUS RCT	Sample: 309 Setting: Single teaching hospital Diagnostic group: Cardiac, Respiratory, Falls, General admission	Inclusion criteria: ≥80 years old; admitted under internal medicine, cardiology or respiratory medicine; English. Exclusion criteria: Not competent; <80 years of age; expected to die or be d/c within 24 hours; had previous ACP; no family.	Type: Received ACP from trained facilitator: multi-disciplinary collaborative approach to ACP; involvement of a surrogate; documentation of EoL care preferences including CPR Control: Usual medical care, no ACP advice unless specifically requested.	Primary: % pts EOL wishes known and respected Secondary: Patient satisfaction; Impact of death on relatives Follow-up: Baseline, death or discharge of patient; 3 and 6 months post death or discharge.	More decedents in intervention groups had EoL wishes known and respected compared to control (86% vs 30%) A greater proportion of family in I group were satisfied with the quality of death of the patient compared to the C group (83% vs 48%)
Gade et al. 2008 [32] RCT	Sample: 517 Setting: Two hospitals Diagnostic group: Mixed Mean age: 73.6 (SD=12.6)	Inclusion criteria: 18+ years, hospitalised with 1+ life-limiting illness, attending physician judgement of prognosis <12 months Excluded: cognitive status impaired, no surrogate or currently enrolled in hospice or PC studies.	Type: Palliative care consultation (IPCS) assessing symptoms, assisting goals of care discussions, discharge planning issues, Control: Not specified	Primary: Symptom control, emotional and spiritual support, satisfaction and health care costs Secondary: Follow-up: 2 weeks discharge, 6 months	No difference in hospital LOS IPCS had longer median hospice stays, fewer ICU admissions Higher % IPCS completed ADs at discharge No difference in symptoms, emotional, spiritual support No difference in survival - more IPCS died during index admission Satisfaction higher for intervention patients Total costs lower by $6766 per patient.
Hanks et al. 2002 [33] UK RCT	Sample: 261 Setting: Hospital Diagnostic group: Cancer and non-malignant inpatients Mean age: 68.4 (26–93)	Inclusion: All inpatient referrals to palliative care team. Exclusion: unable to give consent or complete baseline, unaware of diagnosis, likely to die or be d/c in 24 hours, or needed to be seen very urgently	Type: Full-PCT – assessment by specialist doctor/nurse, provision of advice to team verbally and documented, telephone and in-person follow-up. At least weekly reviews, and liaison with community teams post-discharge. Control: Telephone consultation with senior member of PCT and referring doctor and senior nurse and ward nursing staff	Primary: Symptom control, HrQoL, LOS hospital and rate of re-admission Secondary: Satisfaction patient/family & provider, use of health services. Follow-up: 1 week post-recruitment	Improvement over time in scores for all items in FPCT; and smaller improvements in control No difference between the groups. FPCT discharged home spent fewer days at home, but received more GP visits
Sidebottom et al. 2015 [34] USA RCT	Sample: 232 Response Rate: Setting: Tertiary hospital Diagnostic group: Heart failure Mean age: 73.4 (SD=13.0)	Inclusion criteria: Acute hearty failure, 18+ years. Exclusion: ICU, undergoing evaluation for transplant or LVAD, post-LVAd or transplant, actively dying, cognitive impairments, insufficient English, existing PC order.	Type: PC consult within 24 hour. Differed to usual consult (1) baseline assessment results available to providers; (2) subsequent consults billed to patients. Referral to ACP process at discharge, post-discharge telephone call if ACP not completed. Control: Usual care	Primary: Symptom burden, depressive symptoms, quality of life Secondary: ACP, inpatient 30 day readmission, hospice use, death Follow-up: 1 and 3 months	Intervention: greater reduction in ESAS distress; improvements in SOB, anxiety and tiredness (1 and 3 mths); pain (3 mths only); lower depression score; higher QoL score. Intervention 2.87 times more likely to complete ACP No difference in readmissions, hospice use, death
Song et al. 2005 [35] USA RCT	Sample: 32 dyads Response Rate: Setting: Cardiothoracic surgery clinic Diagnostic group: Cardiac condition requiring surgery Mean age: 69.8 (SD= 8.6)	Inclusion criteria: scheduled for semi-elective surgery in 12 or more hours; had decision-making capacity; >50 years of age; had a surrogate >18 years of age willing to participate.	Type: Patient-Centered Advance Care Planning (PC-ACP) interview (20–45 minutes) by trained nurse: i) representational assessment; ii) exploring concerns planning for future medical decision-making; iii) creating conditions for conceptual change; iv) disease-specific statement of treatment preferences; v) summary. Control: Information card on ADs. Offered more information about AD’s if requested. Pts referred to pastoral counselling at their own request.	Primary: Congruence (measured over 3 scenarios) Secondary: Anxiety (SAI); Pt decision making difficulty (16 item decisional conflict scale); Knowledge of ACP. Follow-up: Baseline and post-interview	Intervention group had significantly higher congruence and lower decisional conflict No other differences.

### Effectiveness of interventions examining end of life processes

Two of three studies reported benefits for providing written or audio-visual information to hospitalized patients on completion of ACDs and CPR orders. Patient who received scripted information about cardio-pulmonary resuscitation (CPR), mechanical ventilation, and ACDs more likely to clarify preferences for treatment and create ACDs [25]. A 3-min video about CPR and intubation improved documentation of CPR orders and intubation, patient knowledge and fewer seriously ill people chose these treatments compared to control patients [21]. However, videotaped interviews and written instructions did not improve ACD rates [20].

More intensive strategies have had mixed success. Smaller studies of provider facilitated advance care planning interventions also reported benefits in surgical [22, 35] and elderly hospitalised patients [24]. Palliative care consultations were found to increase engagement in advance care planning among heart failure and mixed hospitalised populations [32, 34]. A multi-component system-based approach of site visits; a decision support tool; and staff education and training improved the rate of completion of ACDs and some, but not all medical orders [19]. In an ITS trial, completion of ACDs increased significantly during the intervention phase, as did agreement between ACDs and patient preferences [26]. Staff education, dedicated discussion time and increased palliative care involvement increased the rate of documented GOCD and limiting treatment orders [23].

### Effectiveness Intervention studies examining end-of-life outcomes

Two studies examined the impact of facilitated discussions about end-of-life care preferences with patients and support persons. In the SUPPORT trial [30], no significant improvements were found in relation to patient and physician agreement on preferences to withhold resuscitation, pain, hospital resource use or median time until a DNR order was written. However, receiving formal ACP from a trained facilitator improved adherence to wishes; satisfaction, and reduced stress, anxiety, and depression among older inpatients and carers [36]. Three of four studies reported benefits of palliative care consultations on patient outcomes, health care utilisation and costs. Benefits included lower total costs and longer hospice stays [32]; as well as improved symptoms [33, 34]. No significant difference in carer-perceived overall quality of care was found as a consequence of implementing the Liverpool Care Pathway in 16 Italian hospitals [31].

## Discussion

### Volume of research over time and by study type

The growing number of publications in this field reflects the increasing medical and societal demand for improved end-of-life care in hospitals. Given the methodological problems involved in intervention studies, most published studies are descriptive in nature. Many were comprised of retrospective audits examining receipt of life-sustaining treatments, patient symptoms and end-of-life documentation. Others examined patient and family perceptions of care quality or health status. Few were measurement studies, which may reflect the challenges associated with measuring outcomes of effective end-of-life communication. Only 10% of the total were intervention studies.

### Quality of interventions aimed at improving end-of-life processes and outcomes

Only 18 of the 48 intervention studies aimed at improving end of life processes met EPOC design criteria. The remainder were historical control trials, which provide potentially promising data on the feasibility and acceptability of different intervention, but require more rigorous testing. Methodological quality of the included intervention studies was variable. Particular attention needs to be paid to reporting on blinding of outcome assessment and methods of generating allocation sequence and concealing allocation.

### Effectiveness of interventions examining end of life processes and outcomes

Patient-directed interventions represent a less resource intensive approach to increasing the uptake of end-of-life processes. However, the potential reach of these interventions may be limited in hospitalised populations. Unstable patients experiencing acute illness and those lacking capacity comprise a significant proportion of hospitalised populations. This group are unlikely to utilise patient-directed interventions. In these cases, the substitute decision maker may be called on to communicate or make decisions on behalf of patients [3], so would make an appropriate alternative target for intervention. Interventions have also typically focused improving certain end-of-life processes, such as completion of ACDs, without acknowledging the potential role that other processes may play [11]. Segmenting care in this manner does not necessarily mirror the patient’s experience, nor does it recognise that end-of-life care is often synergistic and may require multiple components to be delivered to achieve a positive impact. For instance, introducing a reminder system to increase rates of end-of-life discussions is unlikely to have an impact if patients and staff lack the requisite knowledge and skills to discuss these issues effectively. Hospitals are also made up of individuals with different preferences, skills and motivation to change [6]. Therefore, relying on individuals who are willing and able to be involved in end-of-life research can bias findings. For example, the failure of the landmark SUPPORT trial has been partly attributed to a focus on improving patient-level decision-making without addressing larger, system-related challenges [36].

A more efficient and effective approach may be to support the implementation of system-level changes with potential to benefit everyone within the hospital setting. These approaches allow multiple interventions to be delivered in tandem to address deficits across a range of processes and outcomes. However, they can also pose unique challenges in relation to determining which components contribute to positive change [19]. Adopting alternative research designs, such as multiple baseline and stepped wedge designs has the potential to contribute to the evidence while maintaining methodological rigour [37].

Examining the impact of interventions on end-of-life processes alongside outcomes can provide a balanced picture of healthcare delivery, as it can help to determine whether successful implementation of an end-of-life process positively impacts end-of-life outcomes.

However, the extent to which interventions which target end-of-life processes translate to improved end-of-life outcomes is unclear. Mixed benefits of ACP and palliative care interventions were reported in relation to concordance between preferred and actual care, health status, quality of life and health care costs [11]. These findings are consistent with advance care planning reviews of studies undertaken in other care settings [12]. Reviews of palliative care interventions in ICU settings suggest that consultative approaches, in which palliative care teams consult with the treating team, may be more effective than approaches which attempt to integrate palliative care principles into the daily routines (i.e. integrative approach) [38]. Further research examining this hypothesis is warranted. Given that these interventions often rely on dedicated resources, evidence of effectiveness and sustainability within variable hospital environments must be established.

### Directions for future research

Strategies that intervene with substitute decision makers as well as patients should be explored, given likelihood of impaired capacity among hospitalised patients [39]. In particular, methodological rigorous studies examining multi-faceted, system-based interventions such as education; checklists or tools; audit and feedback and reminders should be undertaken [39]. Future research efforts should also be focused on evaluating consultative palliative care interventions that aim to ensure patients are getting the right care. Further evidence of the benefits for these more complex interventions on end-of-life outcomes, as well as their sustainability must be established.

Introducing topics such as ACP and palliative care in the community may also help alleviate pressure on hospitals. Currently, this is not done in a systematic way [40, 41]. Undertaking ACP in the community may allow preferences to be discussed and decisions made outside the context of a health crisis [42]. Increasing awareness about palliative care may lead to more positive impressions, more equitable uptake of services and improved care quality [43]. While ACP uptake is low among the general public, people are willing to discuss their views about end-of-life issues [44]. General practitioners are well placed to engage in advance care planning as they see a significant proportion of the population and will often have contextual knowledge about individuals [45]. However, lack of skills, difficulties with defining the right moment, and fear of depriving patients of hope are often cited as barriers [46]. Strategies that promote inter-professional collaboration between providers in different care settings, including primary care, hospital and residential aged care facilities, are needed [39]. Few such approaches have been rigorously evaluated.

### Limitations

First, the search strategy may have resulted in publication bias, as we did not include non-published studies or grey literature and there is different terminology used in different countries. Second, the authors excluded studies of provider-directed interventions when an assessment of impact on patient outcomes or processes was not included (e.g. studies that examined the impact of communication skills training interventions on provider knowledge alone). While these interactive education approaches are promising; these outcomes were not the focus and have been examined previously.

## Conclusions

There is a lack of methodologically rigorous studies in this field. Publications examining end-of-life care in hospitals are predominately descriptive in nature, with few rigorous trials of interventions aimed at improving the care of the dying. More high-quality intervention trials in hospitals are required to make clear recommendations about which strategies are most effective in improving end-of-life care processes, and whether these improvements translate to improved end-of-life outcomes. Interventions targeting both the patient and their substitute decision maker, and those strategies with the potential to change practice patterns at a system level should be explored.

## Additional files

Additional file 1: Search terms. (DOCX 14 kb)

Additional file 2: Summary of outcomes. (DOCX 16 kb)

## Abbreviations

ACD: Advance care directive
ACP: Advance care planning
CPR: Cardio-pulmonary resuscitation
DNR: Do-not-resuscitate
EPOC: Effective practice and organisation of care
GOCD: Goals of care discussions
ICU: Intensive care unit
