# Supplementary material for: Improving hospital-based end of life care processes and outcomes: a systematic review of research output, quality and effectiveness
Source: BMC Palliat Care. 2017 May 19;16:34. doi: 10.1186/s12904-017-0204-1 (PMC5438503; doi:10.1186/s12904-017-0204-1)
Supplement: Supplementary file 1 — Search terms. (DOCX 14 kb) [file 12904_2017_204_MOESM1_ESM.docx]

Database: Ovid MEDLINE(R) and Ovid OLDMEDLINE(R) <1946 to Dec 2015 with Daily Update> Search Strategy:

| 1 | Terminal care/ or Palliative care/ or Terminally ill/ or Life support care/ |
| --- | --- |
| 2 | ("end of life" or "end-of-life").tw. |
| 3 | (serious* ill* or critical* ill*).tw. |
| 4 | 1 or 2 or 3 |
| 5 | exp Withholding treatment/ or treatment refusal/ or right to die/ or personal autonomy/ or patient advocacy/ |
| 6 | exp Advance care planning/ or exp Decision making/ |
| 7 | Advance directive.mp. or exp Advance Directives/ |
| 8 | 5 or 6 or 7 |
| 9 | exp Communication/ |
| 10 | Physician patient relations/ or professional family relations/ or professional patient relations/ |
| 11 | 9 or 10 |
| 12 | 4 and 8 and 11 |
| 13 | Outpatients/ or exp community health services/ or ambulatory care/ or exp residential facilities/ or long-term care/ |
| 14 | intensive care.mp. or exp Critical Care/ |
| 15 | 13 or 14 |
| 16 | 12 not 15 |
| 17 | limit 16 to (humans and yr="1990 -2015") |
| 18 | exp child/ or exp infant/ or childhood.mp. or children.mp. |
| 19 | 17 not 18 |
| 20 | limit 19 to (case reports or comment or editorial or letter or news) |
| 21 | 19 not 20 |

Database: EMBASE 1980 to Dec 2015 with Daily Update> Search Strategy:

| 1 | Terminal care/ or Palliative care/ or Terminally ill/ or Life support care/ or Hospice Care/ |
| --- | --- |
| 2 | ("end of life" or "end-of-life").tw. |
| 3 | ("end of life" or "end-of-life").tw. |
| 4 | 1 or 2 or 3 |
| 5 | exp Withholding treatment/ or treatment refusal/ or right to die/ or personal autonomy/ |
| 6 | exp Advance care planning/ |
| 7 | Advance directive.mp. or exp Advance Directives/ |
| 8 | exp Communication/ |
| 9 | Physician patient relations/ or professional family relations/ or professional patient relations/ |
| 10 | 5 or 6 or 7 |
| 11 | 8 or 9 |
| 12 | 4 and 10 and 11 |
| 13 | Outpatients/ or exp community health services/ or ambulatory care/ or exp residential facilities/ or long-term care/ |
| 14 | critical care.mp. or exp intensive care/ |
| 15 | 13 or 14 |
| 16 | 12 not 15 |
| 17 | limit 16 to (humans and yr="1990 -2015") |
| 18 | exp child/ or exp infant/ or childhood.mp. or children.mp. |
| 19 | 17 not 18 |

CINAHL Complete

| **#** | **Query** |
| --- | --- |
| S10 | S10 S7 AND S8 AND S9 |
| S9 | S3 OR S4 OR S5 OR S6 |
| S8 | S1 OR S2 |
| S7 | hospital OR inpatient OR ( hospitalisation or hospitalization ) |
| S6 | advance care planning or advance directives or physician orders for life sustaining treatment or end-of-life care wishes |
| S5 | withholding treatment OR withdrawing treatment OR treatment refusal |
| S4 | physician-patient relations AND (professional-patient relations or nurse-patient relations ) |
| S3 | communication |
| S2 | seriously ill OR critically ill |
| S1 | terminal care or palliative care or end of life or death care |
