# Supplementary material for: Improving hospital-based end of life care processes and outcomes: a systematic review of research output, quality and effectiveness
Source: BMC Palliat Care. 2017 May 19;16:34. doi: 10.1186/s12904-017-0204-1 (PMC5438503; doi:10.1186/s12904-017-0204-1)
Supplement: Supplementary file 2 — Summary of outcomes. (DOCX 16 kb) [file 12904_2017_204_MOESM2_ESM.docx]

Appendix 1: Summary of outcomes addressed in each intervention study meeting EPOC design criteria (n=18)

| **Study** | **ACP*** | **LST order** | **Medicat.**  **order** | **Hospice referral** | **Forgo LST** | **LST** | **Symptom control** | **Survival** | **LOS** | **Concordance** | **Knowledge** | **Satisfaction** | **Cost** | **QoC** | **Re-admission** | **Psycho- social** | **QoL** | **Medication** |
| --- | --- | --- | --- | --- | --- | --- | --- | --- | --- | --- | --- | --- | --- | --- | --- | --- | --- | --- |
| Ahronheim | S+ | P- |  |  | S- | S |  | P- | P- |  |  |  |  |  | P- |  |  |  |
| Bailey | S+ | P- | P+ | S- |  | S- |  |  |  |  |  |  |  |  |  |  |  |  |
| Cugliari | P+ |  |  |  |  |  |  |  |  |  |  |  |  |  |  |  |  |  |
| Constantini | S- |  |  |  |  |  | S+ (SOB) | S- |  |  |  |  |  | P- |  | S- |  | S- |
| Detering | S+ |  |  |  |  |  |  |  |  | P+ |  | S+ (carer) |  |  |  | S+ (carer) |  |  |
| El-Jawahri | P+ | S+ |  |  |  |  |  |  |  |  | S+ |  |  |  |  |  |  |  |
| Gade | S+ |  |  | S- |  |  | P- | S- |  |  |  | P+ (pt) | P+ |  | S+ (ICU) | P- |  |  |
| Grimaldo | P+ |  |  |  |  |  |  |  |  |  |  |  |  |  |  |  |  |  |
| Hanks |  |  |  |  |  |  | P- ǂ |  | P- |  |  | S- (pt) |  |  | P- |  | P- | S- |
| Jacobsen | P+ | P+ |  |  |  |  |  |  |  |  |  |  |  |  |  |  |  |  |
| Meier | P+ |  |  |  |  |  |  |  |  |  |  |  |  |  |  |  |  |  |
| Nicolasara | P+ | P+ |  |  |  |  |  |  |  |  |  |  |  |  |  |  |  |  |
| Reilly | P+ |  |  |  |  |  |  |  |  |  |  |  |  |  |  |  |  |  |
| Sampson | P- |  |  |  |  |  |  |  |  |  |  | S- |  |  |  | S- | S- |  |
| Sidebottom | S+ |  |  | S- |  |  | P+ | S- |  |  |  |  |  |  | S- | P+ | P+ |  |
| Song |  |  |  |  |  |  |  |  |  | P+ (pt-carer) | S- |  |  |  |  | S- |  |  |
| SUPPORT | P- | P- |  |  |  |  | P- |  | P- |  | P- (Dr) |  |  |  |  |  |  |  |
| Teno |  |  |  |  |  |  |  |  |  | P- |  |  |  |  |  |  |  |  |

* may include completion advance directive, appointment health care proxy, and/or discussion of end of life preferences.

ǂ no differences between groups but improved symptoms in both intervention groups over time (no usual care group)

P=primary; S=secondary; + =significant; - =non-significant; QoL=quality of life; LOS=length of stay; LST= life-sustaining treatment; SOB=shortness of breath; ICU=intensive care unit; pt=patient
